# Hydroxychloroquine and Chloroquine Prescribing Patterns by Provider Specialty Following Initial Reports of Potential Benefit for COVID-19 Treatment — United States, January–June 2020

**DOI:** 10.15585/mmwr.mm6935a4

**Published:** 2020-09-04

**Authors:** Lara Bull-Otterson, Elizabeth B. Gray, Daniel S. Budnitz, Heather M. Strosnider, Lyna Z. Schieber, Joseph Courtney, Macarena C. García, John T. Brooks, William R. Mac Kenzie, Adi V. Gundlapalli

**Affiliations:** ^1^Innovation, Technology, and Analytics Task Force, CDC COVID-19 Emergency Response Team; ^2^Division of Health Informatics and Surveillance, Center for Surveillance, Epidemiology, and Laboratory Services, CDC; ^3^Division of Healthcare Quality Promotion, National Center for Emerging and Zoonotic Infectious Diseases, CDC; ^4^Health System and Worker Safety Task Force, CDC COVID-19 Emergency Response Team; ^5^Division of Environmental Health Science and Practice, National Center for Environmental Health, CDC; ^6^Division of Overdose Prevention, National Center for Injury Prevention and Control, CDC; ^7^Office of the Director, Center for Surveillance, Epidemiology, and Laboratory Services, CDC; ^8^Chief Medical Officer, CDC COVID-19 Response Team; ^9^Public Health Informatics Office, Center for Surveillance, Epidemiology, and Laboratory Services, CDC.

Hydroxychloroquine and chloroquine, primarily used to treat autoimmune diseases and to prevent and treat malaria, received national attention in early March 2020, as potential treatment and prophylaxis for coronavirus disease 2019 (COVID-19) (*1*). On March 20, the Food and Drug Administration (FDA) issued an emergency use authorization (EUA) for chloroquine phosphate and hydroxychloroquine sulfate in the Strategic National Stockpile to be used by licensed health care providers to treat patients hospitalized with COVID-19 when the providers determine the potential benefit outweighs the potential risk to the patient.* Following reports of cardiac and other adverse events in patients receiving hydroxychloroquine for COVID-19 (*2*), on April 24, 2020, FDA issued a caution against its use^†^ and on June 15, rescinded its EUA for hydroxychloroquine from the Strategic National Stockpile.^§^ Following the FDA’s issuance of caution and EUA rescindment, on May 12 and June 16, the federal COVID-19 Treatment Guidelines Panel issued recommendations against the use of hydroxychloroquine or chloroquine to treat COVID-19; the panel also noted that at that time no medication could be recommended for COVID-19 pre- or postexposure prophylaxis outside the setting of a clinical trial (*3*). However, public discussion concerning the effectiveness of these drugs on outcomes of COVID-19 (*4*,*5*), and clinical trials of hydroxychloroquine for prophylaxis of COVID-19 continue.^¶^ In response to recent reports of notable increases in prescriptions for hydroxychloroquine or chloroquine (*6*), CDC analyzed outpatient retail pharmacy transaction data to identify potential differences in prescriptions dispensed by provider type during January–June 2020 compared with the same period in 2019. Before 2020, primary care providers and specialists who routinely prescribed hydroxychloroquine, such as rheumatologists and dermatologists, accounted for approximately 97% of new prescriptions. New prescriptions by specialists who did not typically prescribe these medications (defined as specialties accounting for ≤2% of new prescriptions before 2020) increased from 1,143 prescriptions in February 2020 to 75,569 in March 2020, an 80-fold increase from March 2019. Although dispensing trends are returning to prepandemic levels, continued adherence to current clinical guidelines for the indicated use of these medications will ensure their availability and benefit to patients for whom their use is indicated (*3*,*4*), because current data on treatment and pre- or postexposure prophylaxis for COVID-19 indicate that the potential benefits of these drugs do not appear to outweigh their risks.

Hydroxychloroquine and chloroquine prescriptions dispensed through outpatient retail pharmacies in the United States during January–June 2019 and January–June 2020 were examined using deidentified pharmacy transactions from the IQVIA National Prescription Audit database.** This database includes 92% of all outpatient retail prescriptions dispensed in the United States; prescription estimates were projected by IQVIA to represent all retail outpatient medication dispensing at the state and national levels.

New prescriptions for hydroxychloroquine and chloroquine were defined as those dispensed to a patient without a history of prescription for these medications in the preceding 12 months. Hydroxychloroquine accounted for approximately 99% of prescriptions dispensed during the study period. Refill/switch prescriptions were defined as those dispensed either as a refill of a previous prescription or as a new prescription with a change in medication strength or brand or switches between medications within the same therapeutic category (i.e., bidirectional switches of hydroxychloroquine and chloroquine). New and refill/switch prescriptions dispensed before reports of potential benefit on medication use for COVID-19 (during January–June 2019) were compared with new and refill/switch prescriptions during January–June 2020. Fold changes in the numbers of new prescriptions were calculated and defined as the ratio between the estimated number of prescriptions in March, April, May, and June 2020, with respect to the same months in 2019. The percentage of total dispensed prescriptions by specialty group was calculated using the total number of dispensed prescriptions by specialty group, divided by the overall total number of dispensed prescriptions for the month; the percentage of new prescriptions by a specialty group was calculated by dividing the new prescriptions dispensed for the specialty group by the total prescriptions for the specialty group. The percentage of new prescriptions dispensed to males was calculated as the number of new prescriptions for males divided by the total number of new prescriptions.

Prescriptions were not included if they were dispensed by mail order; mail-dispensed prescriptions accounted for <7.5% of dispensed hydroxychloroquine and chloroquine. Prescriptions by veterinarians were also excluded.

Prescriptions included information on the prescriber’s medical specialty, as defined by the American Medical Association (AMA) self-designated practice specialties.^††^ For this study, clinicians prescribing hydroxychloroquine or chloroquine were categorized based on the frequency of prescribing of hydroxychloroquine or chloroquine before the COVID-19 pandemic. Specialists from rheumatology, dermatology, allergy, and nephrology, who might have had experience using these drugs for indicated medical conditions within their specialty before the pandemic (collectively termed routine prescribers) were responsible for 62% of new hydroxychloroquine or chloroquine prescriptions in 2019. Allopathic and osteopathic physicians, who included internal medicine, family practice, general practice, and pediatrics, and nurse practitioners, physician assistants, and prescribers with unspecified specialty (per AMA classification) were grouped for this study into primary care prescribers; this group provided 35% of the new prescriptions in 2019. Other specialists were considered nonroutine prescribers^§§^ if, in 2019, their specialty prescribed ≤2% of hydroxychloroquine or chloroquine prescriptions. Nonroutine prescribing specialties are less likely under normal circumstances to directly manage patients with autoimmune disorders or provide prescriptions for malaria prophylaxis.

The overall estimated number of hydroxychloroquine or chloroquine prescriptions dispensed in March and April 2020 increased from 819,906 in 2019 to 1,312,859 in 2020 (Table). In 2019, 92% of prescriptions were refill/switch prescriptions. Refill/switch prescriptions increased 1.4-fold, from 377,222 in March 2019 to 536,804 in March 2020, and remained elevated in April (456,489; 1.2-fold higher than in April 2019) (Figure 1). New prescriptions for hydroxychloroquine or chloroquine in March 2020 (222,382) were 7.2-fold higher than the 30,737 prescriptions in March 2019; in April, the number of new prescriptions (106,184) was 3.3-fold higher than the 31,748 in April 2019 (Table).

**TABLE:** Estimated hydroxychloroquine or chloroquine retail dispensing, by prescriber category — United States, January–June, 2019 and 2020*

Specialty/Prescription characteristic	2019						2020					
	Jan	Feb	Mar	Apr	May	June	Jan	Feb	Mar	Apr	May	June
**All providers (routine, primary care or unspecified, and nonroutine)**												
No. of total prescriptions.	414,278	373,985	407,959	411,947	420,901	396,620	413,345	383,435	759,186	562,673	474,360	500,473
Refill/Switch prescriptions^†^	383,105	345,244	377,222	380,199	387,761	366,750	381,260	352,959	536,804	456,489	436,823	461,670
Fold change in refill/switch prescriptions from 2019	—	—	—	—	—	—	1.0	1.0	1.4	1.2	1.1	1.3
New prescriptions, no. (% of total)	31,173 (7.5)	28,741 (7.7)	30,737 (7.5)	31,748 (7.7)	33,140 (7.9)	29,871 (7.5)	32,085 (7.8)	30,476 (7.9)	222,382 (29.3)	106,184 (18.9)	37,537 (7.9)	38,803 (7.8)
New prescriptions for males, no. (% new)	6,049 (19.4)	5,495 (19.1)	5,834 (19.0)	5,960 (18.8)	6,393 (19.3)	5808 (19.4)	5,791 (18)	5,664 (18.6)	93,776 (42.2)	40,055 (37.7)	9,916 (26.4)	9213 (23.7)
Fold change new prescriptions from 2019	—	—	—	—	—	—	1.0	1.1	7.2	3.3	1.1	1.3
% New prescriptions from combined primary care or routine specialty	96.9	97.0	97.0	97.1	96.8	96.9	97.4	96.2	66.0	84.3	94.0	94.9
Routine prescribers**												
% of total prescriptions	64.1	64.2	64.6	64.7	64.9	64.9	64.2	64.1	49.7	53.9	61.5	62.1
No. of prescriptions	265,495	240,259	263,559	266,599	273,155	257,508	265,571	245,842	377,271	303,253	291,741	310,839
Refill/Switch prescriptions^†^	246,518	222,477	244,101	246,401	252,400	238,899	245,640	227,261	339,163	280,823	274,218	290,907
Fold change in refill/switch prescriptions from 2019	—	—	—	—	—	—	1.0	1.0	1.4	1.1	1.1	1.2
New prescriptions, no. (% in-group total)	18,977 (7.1)	17,782 (7.4)	19,458 (7.4)	20,198 (7.6)	20,755 (7.6)	18,609 (7.2)	19,931 (7.5)	18,581 (7.6)	38,108 (10.1)	22,430 (7.4)	17,523 (6.0)	19,932 (6.4)
New prescriptions for males, no. (% new)	3,279 (17.3)	3,074 (17.3)	3,398 (17.5)	3,488 (17.3)	3,590 (17.3)	3290 (17.7)	3,276 (16.4)	3,067 (16.5)	9,559 (25.1)	4,292 (19.1)	3,143 (17.9)	3,518 (17.6)
Fold change in new prescriptions from 2019	—	—	—	—	—	—	1.1	1.0	2.0	1.1	0.9	1.1
**Primary care or unspecified specialty prescribers^¶^**												
% of total prescriptions	33.9	33.7	33.4	33.3	33.1	33.1	33.9	33.9	38.2	40.6	35.9	35.5
No. of prescriptions	140,386	126,216	136,376	137,242	139,124	131,153	140,090	130,024	290,277	228,584	170,469	177,664
Refill/switch prescriptions^†^	129,164	116,131	126,026	126,616	127,805	120,830	128,768	119,272	181,572	161,529	152,703	160,767
Fold change in refill/switch prescriptions from 2019	—	—	—	—	—	—	1.0	1.0	1.4	1.3	1.2	1.3
New prescriptions, no. (% in-group total)	11,222 (8.0)	10,085 (8.0)	10,350 (7.6)	10,626 (7.7)	11,319 (8.1)	10,323 (7.4)	11,322 (8.1)	10,752 (8.3)	108,705 (37.4)	67,055 (29.3)	17,766 (10.4)	16,897 (9.5)
New prescriptions for males, no. (% new)	2,494 (22.2)	2,189 (21.7)	2,194 (21.2)	2,239 (21.1)	2,486 (22.0)	2256 (21.8)	2,322 (20.5)	2,211 (20.6)	48,283 (44.4)	27,978 (41.7)	5,838 (32.9)	4,931 (29.2)
Fold change in new prescriptions from 2019	—	—	—	—	—	—	1.0	1.1	10.5	6.3	1.6	1.6
**Nonroutine prescribers^§^**												
% of total prescriptions	2.0	2.0	2.0	2.0	2.1	2.0	1.9	2.0	12.1	5.5	2.6	2.4
No. of prescriptions	8,397	7,510	8,024	8,107	8,622	7,960	7,684	7,569	91,639	30,836	12,150	11,970
Refill/Switch prescriptions^†^	7,423	6,636	7,095	7,183	7,556	7,021	6,852	6,426	16,070	14,137	9,902	9,996
Fold change in refill/switch prescriptions from 2019	—	—	—	—	—	—	0.9	1.0	2.3	2.0	1.3	1.4
New prescriptions, no. (% in-group total)	974 (11.6)	874 (11.6)	929 (11.6)	924 (11.4)	1,066 (12.4)	939 (11.8)	832 (10.8)	1,143 (15.1)	75,569 (82.5)	16,699 (54.2)	2,248 (18.5)	1,974 (16.5)
New prescriptions for males, no. (% new)	275 (28.2)	232 (26.5)	242 (26.0)	233 (25.2)	317 (29.7)	263 (28.0)	193 (23.2)	386 (33.8)	35,934 (47.6)	7785 (46.6)	934 (41.5)	765 (38.7)
Fold change in new prescriptions from 2019	—	—	—	—	—	—	0.9	1.3	81.3	18.1	2.1	2.1

* Prescription data for 2017 and 2018 were also examined but found consistent with 2019, without remarkable month to month variation.

^†^ Refill/switch prescriptions include dispensed prescriptions that were either a refill or a new prescription for a different dose or a switch in brand.

^§^ Nonroutine = addiction medicine, allergy/immunology, anesthesiology, cardiology, cardiothoracic surgery, cardiovascular surgery, clinical neurophysiology, clinical pharmacology, colon and rectal surgery, critical care, critical care medicine, dentistry, dermatopathology, diagnostic laboratory, diagnostic laboratory immunology, emergency medicine, endocrinology, gastroenterology, general preventive medicine, general surgery, genetics, geriatric psychiatry, geriatrics, hematology, hepatology, hospice and palliative medicine, infectious disease, medical microbiology, naturopathic doctor, neurologic surgery, neurology, neurosurgery-critical care, nuclear medicine, nutrition, obstetrics/gynecology, obstetrics/gynecology-critical care, occupational medicine, oncology, ophthalmology, optometry, orthopedic surgery, orthopedic surgery of spine, other, other surgery, otolaryngology, otology, pain medicine, pathology, pediatric critical care, pediatric neurosurgery, pharmacist, physical medicine and rehab, plastic surgery, podiatry, psychiatry, psychology, pulmonary critical care, pulmonary diseases, radiology, sleep medicine, sports medicine, surgery, thoracic surgery, and urology.

^¶^ Primary care/unspecified = family practice, general practice, internal medicine, internal medicine/pediatrics, nurse practitioner, osteopathic medicine, pediatrics, physician assistant, and specialty unspecified.

** Routine = allergy, dermatology, nephrology, and rheumatology.

**FIGURE 1:**
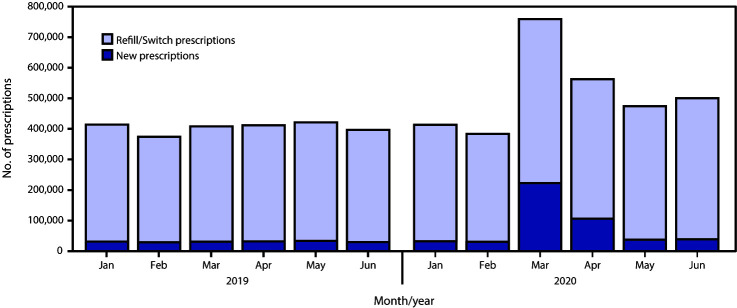
Estimated refill/switch* and new retail prescriptions for hydroxychloroquine or chloroquine dispensed in the United States — January–June, 2019–2020 * Refill/switch prescriptions include dispensed prescriptions that were either a refill of an existing prescription or a new prescription for a different dose or a brand switch.

Overall, 54% of new prescriptions in March and April 2020 were written by primary care prescribers. In March 2020, primary care prescribers wrote more new prescriptions than did routine prescribers, writing 10,350 dispensed prescriptions in 2019 compared with 108,705 in 2020, a 10.5-fold increase (Figure 2). Primary care prescribers continued to be the largest source of new prescriptions in April 2020, writing 67,055 prescriptions (63% of total new prescriptions).

**FIGURE 2:**
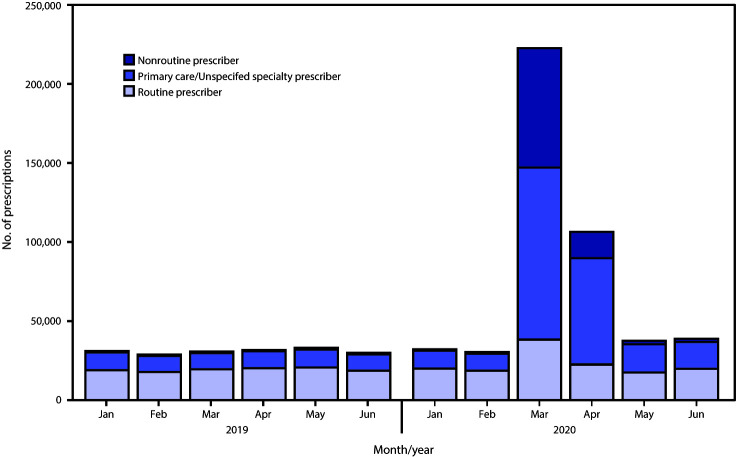
Estimated new retail prescriptions of hydroxychloroquine or chloroquine dispensed, by prescriber category* — United States, January–June, 2019–2020 * *Nonroutine prescribers* = addiction medicine, allergy/immunology, anesthesiology, cardiology, cardiothoracic surgery, cardiovascular surgery, clinical neurophysiology, clinical pharmacology, colon and rectal surgery, critical care, critical care medicine, dentistry, dermatopathology, diagnostic laboratory, diagnostic laboratory immunology, emergency medicine, endocrinology, gastroenterology, general preventive medicine, general surgery, genetics, geriatric psychiatry, geriatrics, hematology, hepatology, hospice and palliative medicine, infectious disease, medical microbiology, naturopathic doctor, neurological surgery, neurology, neurosurgery-critical care, nuclear medicine, nutrition, obstetrics/gynecology, obstetrics/gynecology-critical care, occupational medicine, oncology, ophthalmology, optometry, orthopedic surgery, orthopedic surgery of spine, other, other surgery, otolaryngology, otology, pain medicine, pathology, pediatric critical care, pediatric neurosurgery, pharmacist, physical medicine and rehab, plastic surgery, podiatry, psychiatry, psychology, pulmonary critical care, pulmonary diseases, radiology, sleep medicine, sports medicine, surgery, thoracic surgery, and urology. *Primary care/unspecified prescribers* = family practice, general practice, internal medicine, internal medicine/pediatrics, nurse practitioner, osteopathic medicine, pediatrics, physician assistant, and specialty unspecified. *Routine prescribers* = allergy, dermatology, nephrology, and rheumatology.

During March and April 2020, nonroutine prescribers accounted for the largest percentage increase in new prescriptions compared with the same period in 2019 (81.3-fold and 18.1-fold increases in March and April, respectively). The nonroutine prescribing specialties with the highest prescribing volume and growth in March 2020 were ophthalmology, anesthesiology, and cardiology.

During March and April 2019, most new prescriptions were dispensed to females (81%). In 2020, the estimated number of total new prescriptions for males was 93,776 in March (16.1-fold higher than March 2019), and 40,055 in April (6.8-fold higher than April 2019), accounting for 42% and 38% of all new prescriptions in March and April, respectively.

In May and June 2020, refill/switch prescriptions declined but remained elevated: 436,823 in May (1.1-fold higher than May 2019) and 461,670 in June (1.3-fold higher than June 2019). New prescriptions in May 2020 declined to 37,537 (7.9%) of all dispensed hydroxychloroquine or chloroquine prescriptions, with a similar number of dispensed prescriptions (38,803; 7.8%) in June 2020. In May 2020, the percentage of new prescriptions by those in nonroutine prescribing specialties declined to 18.5% from 82.5% in March and 54.2% in April.

## Discussion

In the United States, during March and April 2020, monthly hydroxychloroquine and chloroquine outpatient prescribing was higher than it was during the previous year. These medications are routinely prescribed for lupus and rheumatoid arthritis (hydroxychloroquine) and for antimalarial prophylaxis malaria treatment (chloroquine), and the annual rate of prescribing has not varied substantially from year to year (*6*). In contrast, new prescriptions written by primary care prescribers and nonroutine prescribing specialists increased significantly in 2020. Primary care prescribers provided 54% of new prescriptions dispensed at outpatient retail pharmacies during March–April 2020; the largest percentage increase in new prescriptions compared with the same period in 2019 was among nonroutine prescribers.

A large increase in new prescriptions occurred for adult males (16.1-fold increase in March 2020 compared with March 2019). This increase in hydroxychloroquine prescribing for males is notable given that females are historically more likely to receive a new hydroxychloroquine prescription for autoimmune disease, consistent with described prevalence of autoimmune disorders among females (78%) (*7*). By May and June 2020, the numbers of new prescriptions and the number of new prescriptions from nonroutine prescribing specialties had declined and were approaching those of 2019. These declines might have been influenced by publication of additional studies indicating that the medications were not found to be effective for treatment of COVID-19 and by FDA safety warning (*8*).

The findings in this report are subject to at least four limitations. First, mail-order prescriptions were not included in the study, nor were prescriptions given in inpatient settings, so data do not indicate total medication use nationwide. However, the data are weighted to be nationally representative, although they are based on a sample of 92% of outpatient prescriptions. Second, because specialty information was lacking for nurse practitioners, physician assistants, and unspecified specialties, these prescribers were categorized as primary care; however, it is possible that these providers were working in routine or nonroutine prescriber practices. In addition, allopathic and osteopathic physicians with internal medicine and subspecialty training potentially were not classified by subspecialty. Third, among patients receiving prescriptions, clinical indications, patients’ underlying medical conditions, and concurrent medications were unknown. Finally, no information was available to confirm whether the medication was taken or stored for future use or if any adverse events occurred.

If prescribing or prescribed these drugs, providers and patients should be familiar with the potential for drug interactions and adverse events associated with hydroxychloroquine or chloroquine use (*8*,*9*). The importance of obtaining a patient’s complete medical and medication history to evaluate risks should be emphasized to nonroutine prescribers of hydroxychloroquine or chloroquine. In the setting of polypharmacy and comorbid conditions, such as preexisting heart conditions, performing an electrocardiogram to evaluate the QT interval before starting these medications is advisable, because hydroxychloroquine or chloroquine can prolong the QT interval, leading to malignant arrhythmias such as torsade de pointes or ventricular fibrillation (*9*). Because of the long-terminal half-life of hydroxychloroquine (>40 days) (*10*), patients could continue to be at risk for drug interactions and adverse cardiac events after the course of therapy is completed.

Although federal guidelines now recommend against using hydroxychloroquine or chloroquine for the treatment or prevention of COVID-19, dispensing policies and restrictions vary significantly by state (*8*). Policies by boards of pharmacy in some states, such as New Jersey, require hydroxychloroquine prescriptions to include a diagnosis, documentation of a positive diagnostic test, and be limited to a 14-day supply.^¶¶^ In Texas, similar restrictions instituted in May expired in July.*** Several other states advise caution in prescribing hydroxychloroquine or chloroquine for COVID-19, while allowing for clinical judgement without policy limitations. Although dispensing of hydroxychloroquine or chloroquine prescriptions has been declining since March 2020, continued attention to updated clinical guidance (*3*,*4*), especially by nonroutine prescribers, will help safeguard supplies and ensure safe use of these medications for patients with approved indications.^†††^

**Summary**

**What is already known about this topic?**

Hydroxychloroquine and chloroquine are approved to treat autoimmune diseases and to prevent and treat malaria. Earlier this year, they were widely reported to be of potential benefit in the prevention and treatment of COVID-19; however, current data indicate that the potential benefits of these drugs do not outweigh their risks.

**What is added by the report?**

New prescriptions by specialists who did not typically prescribe these medications (defined as specialties accounting for ≤2% of new prescriptions before 2020) increased from 1,143 prescriptions in February 2020 to 75,569 in March 2020, an 80-fold increase from March 2019.

**What are the implications for public health practice?**

Attention to updated clinical guidance, especially by nonroutine prescribers, will help safeguard supplies and ensure safe use of hydroxychloroquine and chloroquine for patients with approved indications.

## Footnotes

* https://www.fda.gov/media/136534/download.

† https://www.fda.gov/drugs/drug-safety-and-availability/fda-cautions-against-use-hydroxychloroquine-or-chloroquine-covid-19-outside-hospital-setting-or.

§ https://www.fda.gov/media/138945/download.

¶ https://www.ncbi.nlm.nih.gov/pmc/articles/PMC7330261/.

** IQVIA projected prescription estimates using proprietary methods and information internal to the company. https://www.iqvia.com/locations/united-states/solutions/commercial-operations/essential-information/prescription-information.

†† http://www.dmddata.com/2009_05_sdps.pdf.

§§ Nonroutine specialties included addiction medicine, allergy/immunology, anesthesiology, cardiology, cardiothoracic surgery, cardiovascular surgery, clinical neurophysiology, clinical pharmacology, colon and rectal surgery, critical care, critical care medicine, dentistry, dermatopathology, diagnostic laboratory, diagnostic laboratory immunology, emergency medicine, endocrinology, gastroenterology, general preventive medicine, general surgery, genetics, geriatric psychiatry, geriatrics, hematology, hepatology, hospice and palliative medicine, infectious disease, medical microbiology, naturopathic doctor, neurologic surgery, neurology, neurosurgery-critical care, nuclear medicine, nutrition, obstetrics/gynecology, obstetrics/gynecology-critical care, occupational medicine, oncology, ophthalmology, optometry, orthopedic surgery, orthopedic surgery of spine, other, other surgery, otolaryngology, otology, pain medicine, pathology, pediatric critical care, pediatric neurosurgery, pharmacist, physical medicine and rehab, plastic surgery, podiatry, psychiatry, psychology, pulmonary critical care, pulmonary diseases, radiology, sleep medicine, sports medicine, surgery, thoracic surgery, and urology.

¶¶ https://www.nj.gov/oag/newsreleases20/DCA_AO_2020-01.pdf.

*** https://www.pharmacy.texas.gov/files_pdf/291.30.pdf.

††† https://www.accessdata.fda.gov/scripts/drugshortages/default.cfm.
